# Safety and visual outcomes following posterior chamber phakic intraocular lens bilensectomy

**DOI:** 10.1186/s40662-020-00200-8

**Published:** 2020-07-01

**Authors:** Veronica Vargas, Jorge L. Alió, Rafael I. Barraquer, Justin Christopher D’ Antin, Cristina García, Francisco Duch, Joan Balgos, Jorge L. Alió del Barrio

**Affiliations:** 1 Vissum Instituto Oftalmológico de Alicante, Alicante, Spain; 2 Division of Ophthalmology, Universidad Miguel Hernández, Alicante, Spain; 3 Institut Universitari Barraquer, Universitat Autònoma de Barcelona, Barcelona, Spain; 4 Centro de Oftalmología Barraquer, Barcelona, Spain; 5 Universitat Internacional de Catalunya, Barcelona, Spain; 6 Clínica Real Vision, Madrid, Spain; 7 Instituto Catalán de Retina (ICR) unidad de Cirugía Refractiva, Barcelona, Spain

**Keywords:** Bilensectomy, Posterior chamber phakic intraocular lenses, Cataract, Endothelial cell count, Visual outcomes, Postoperative complications, Intraoperative complications

## Abstract

**Background:**

To evaluate the safety, efficacy, refractive outcomes and causes for bilensectomy (phakic intraocular lens – pIOL – explantation with cataract surgery and pseudophakic intraocular lens implantation) in patients previously implanted with posterior chamber pIOLs.

**Methods:**

This multi-center retrospective study included 87 eyes of 55 patients who underwent bilensectomy for posterior chamber pIOL with a follow up time of 12 months. The uncorrected and best corrected distance visual acuities (UDVA, CDVA), endothelial cell density before and after bilensectomy were assessed, as well as the cause of bilensectomy and intra or postoperative complications.

**Results:**

There was a statistically significant improvement in uncorrected and best corrected visual acuities after bilensectomy (*p* = 0.00). The main reason for bilensectomy was cataract development (93.1% of the cases), followed by miscalculation of lens size, and corneal edema. The endothelial cell count remained stable without a statistically significant change after surgery (*p* = 0.67). The refractive efficacy index was 0.8, none of the patients lost lines of CDVA after surgery, 73% of the patients were within ±1 D (spherical equivalent) of the target refraction. Intraoperative complications were one posterior capsule rupture with the intraocular lens (IOL) implanted in the sulcus, and 3 eyes required the use of pupil expanders for adequate pupil dilation. Postoperatively, one eye developed retinal detachment. The three pIOLs models explanted were the Implantable Collamer Lens (ICL), Implantable Phakic Contact Lens (IPCL) and the Phakic Refractive Lens (PRL).

**Conclusions:**

Good safety and visual outcomes were observed 1 year after bilensectomy for posterior chamber phakic intraocular lenses (PC pIOLs). There were few intra and postoperative complications and there was no significant endothelial cell loss after the bilensectomy procedure.

## Background

The correction of high ametropias with phakic intraocular lenses (pIOL) has the advantage of excellent visual outcomes, accommodation maintenance, and reversibility; contrary to laser refractive surgery [1–4]. Posterior chamber phakic intraocular lenses (PC pIOLs) are widely used, and their implantation is relatively easy. Furthermore, they have long-term predictable and stable results for the correction of myopia [1], hyperopia [2] and astigmatism [3, 4]. Nowadays, the commercially available PC pIOLs are the Implantable Collamer Lens (Staar Surgical Co, Monriva, California) and the Implantable Phakic Contact Lens (IPCL, Care Group Sight solutions, India). Other PC pIOLs like the phakic refractive lens (PRL, Zeiss Meditec, Jena, Germany) were phased out from the market due to associated long term complications [5].

In spite of all the possible advantages that a pIOL may offer, all patients with a pIOL will eventually undergo bilensectomy (pIOL explantation with cataract surgery and posterior chamber intraocular lens – IOL – implantation), either due to a pIOL-induced cataract or the development of an age-related cataract. Many studies have reported the causes and incidence of cataract after the implantation of a PC pIOL [6–8], but few have really reported the clinical outcomes after bilensectomy [9], with only a few clinical cases available [10, 11]. The aim of this study is to evaluate the safety, efficacy, refractive outcomes, and indications for bilensectomy in patients with posterior chamber pIOLs, with a minimum postoperative follow up of 12 months. To the best of our knowledge, this is the only study that describes the outcomes of bilensectomy with different models of posterior chamber pIOLs.

## Methods

This is a retrospective, multicenter study, involving 87 eyes that had bilensectomy after PC pIOL implantation. All data was obtained from the IBERIA biobank.^1^ The Spanish centers that participated in this study were: Vissum Alicante (Alicante), and Vissum Madrid (Madrid), Centro de Oftalmología Barraquer (Barcelona), and the Instituto Catalán de Retina-ICR (Barcelona). This study was approved by the local research ethics committees and was performed in compliance with the principles of the Declaration of Helsinki.

Indications for the implantation of PC pIOLs were the following: ametropia that could not be corrected with corneal laser refractive surgery (extremely high myopes or patients with thin corneas), anterior chamber depth greater than 2.8 mm, irido-corneal angle greater than 30°, endothelial cell count (ECC) > 2500 cells/mm^2^_._

Indications for bilensectomy were the following: cataract development with loss of two or more lines of corrected distance visual acuity (CDVA) or ECC < 1200 cells/mm^2^.

Preoperative assessment included: uncorrected distance visual acuities (UDVA) and CDVA respectively measured with ETDRS charts, slit lamp examination, Goldmann applanation tonometry, fundus examination, and central ECC measurements taken with a noncontact specular microscope (Noncon Robo-CA, Konan). The intraocular lens (IOL) to be implanted was calculated preoperatively using interferometry (IOLMaster, Carl Zeiss Meditec AG). The SRK-T formula with the Wang/Koch adjustment was used and the target refraction was emmetropia in all cases. Bilensectomy was performed by four experienced surgeons (JLA, JIB, RIB, FD). A monofocal or multifocal IOL was implanted depending on patients’ postoperative visual expectations, preoperative ophthalmic examination, daily activities (intermediate and near visual needs), and age. Toric IOLs were implanted in those patients with a topographic astigmatism > 1.5 D.

The technique for pIOL removal was as follows: Two 1 mm side ports were created, intracameral mydriatic was used to dilate the pupil and dispersive viscoelastic (Viscoat, Alcon) was injected to protect the corneal endothelium. A 3.2 mm clear corneal incision was made. The 4 footplates of the pIOL were carefully lifted into the anterior chamber and carefully explanted through the main incision. Coaxial phacoemulsification was performed with implantation of a posterior chamber IOL. A cohesive viscoelastic (Provisc, Alcon) was used to keep the capsular bag open for IOL insertion. After injecting intracameral antibiotic, the main incision and paracentesis were hydrated and sealed (Additional file 1). Postoperative medications included topical antibiotics for a week and steroids tapered over 4 weeks.


Additional file 1: **Video S1**


The main outcome measures were: efficacy (UDVA after bilensectomy / CDVA after bilensectomy), percentage of eyes in which the postoperative CDVA was worse than the preoperative CDVA (safety), refractive predictability and central ECC change. The secondary outcomes were: bilensectomy etiology, and intra/postoperative complications.

### Statistical analysis

Statistical analysis was performed with the SPSS software for Windows (version 15.0.1). The average values and standard deviations were calculated for every parameter. Normality of all data was evaluated by means of the Kolmogorov-Smirnov test. When parametric analysis was possible, the Student’s t test for paired data was performed for all parameter comparison between preoperative and postoperative examinations. When parametric analysis was not possible, the Wilcoxon test was applied to assess the significance of differences between preoperative and postoperative data. For all statistical tests a *p* value less than 0.05 was considered statistically significant.

## Results

**Fig. 1:**
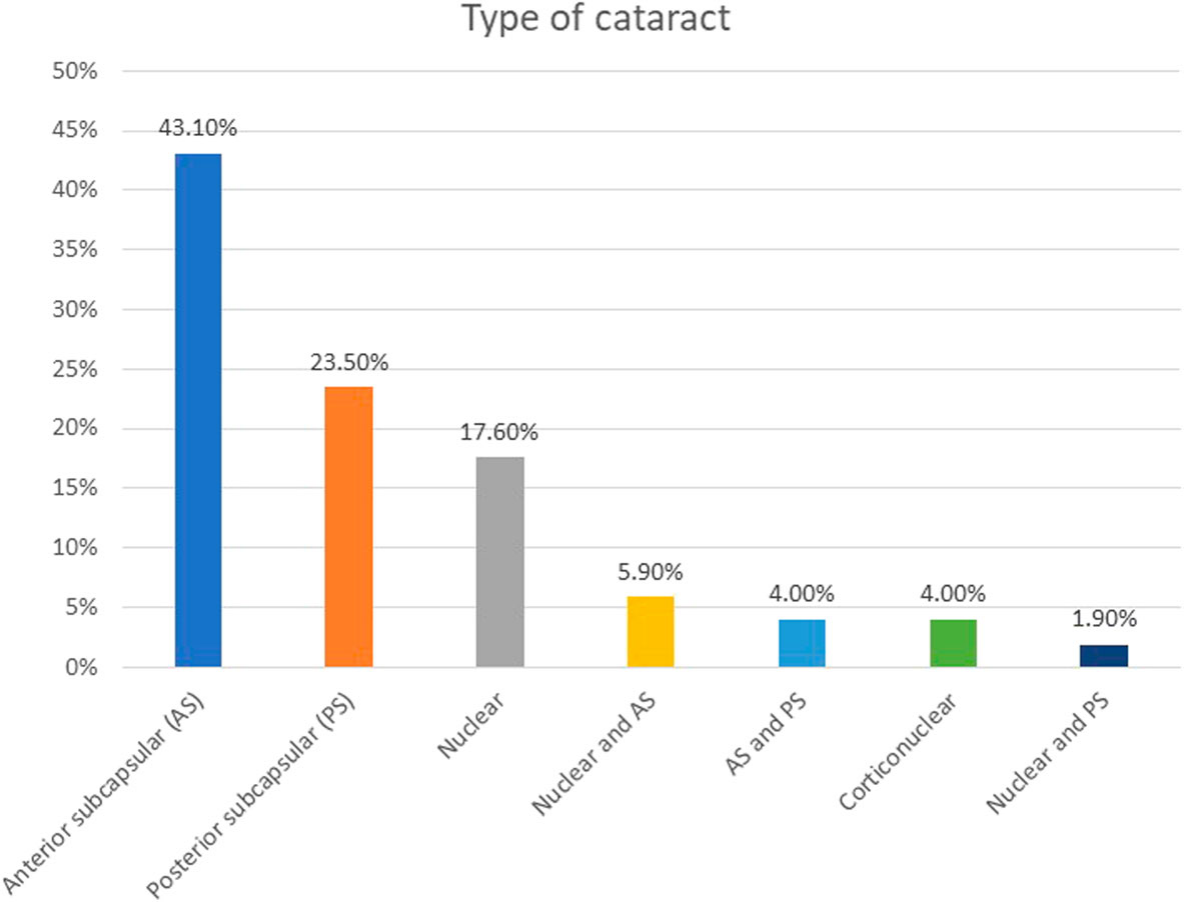
Percentage of the different types of cataracts presented in our study

**Fig. 2:**
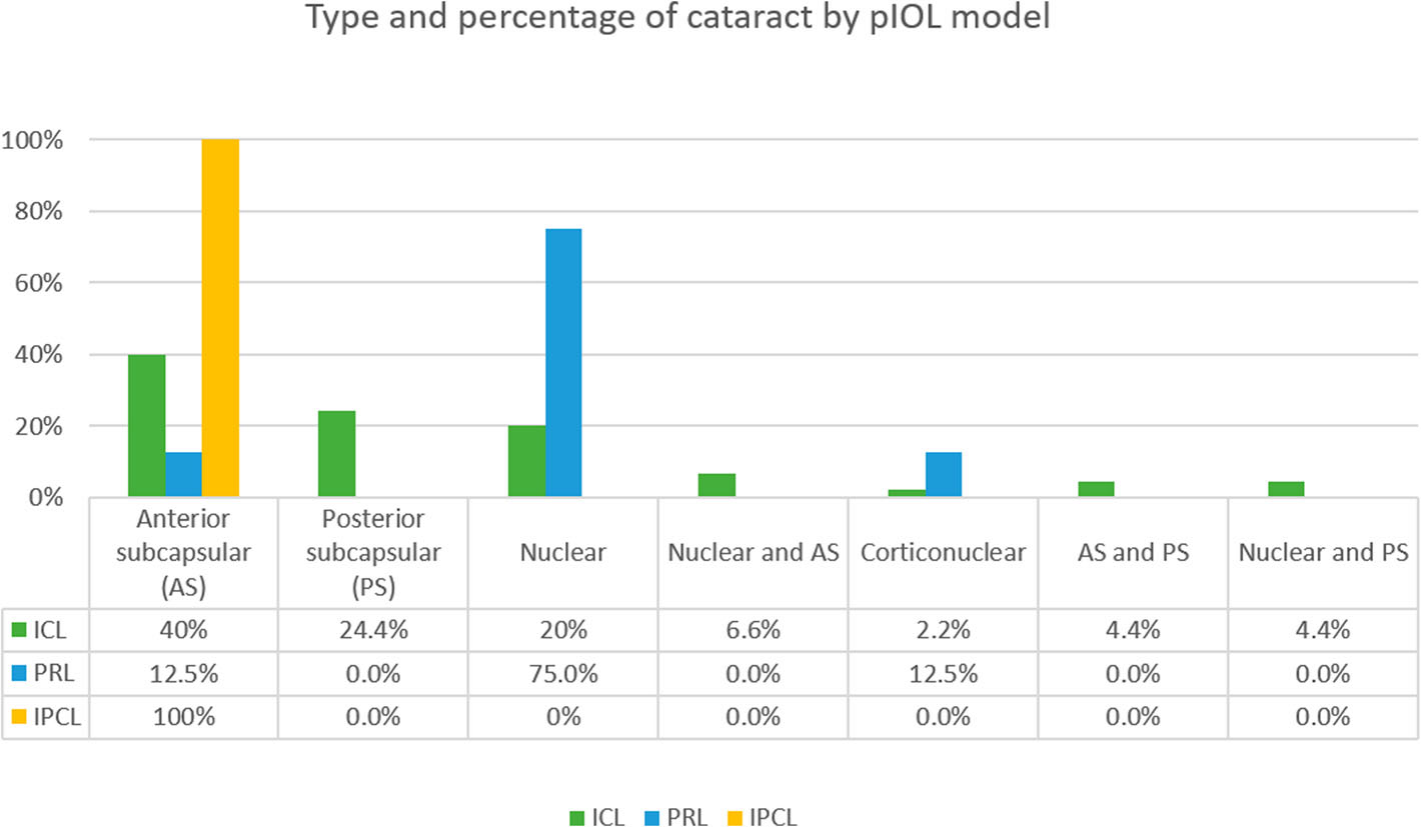
Type and percentage of cataracts developed by each phakic IOL model

**Fig. 3:**
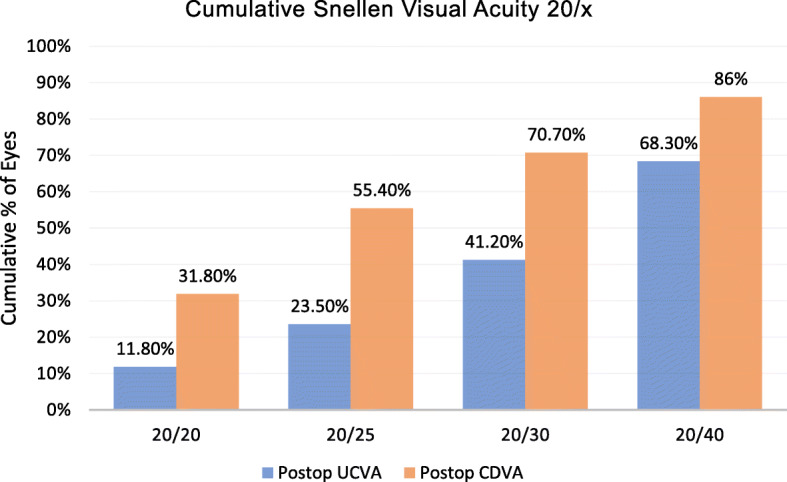
Histogram of postoperative CDVA and UCVA

**Fig. 4:**
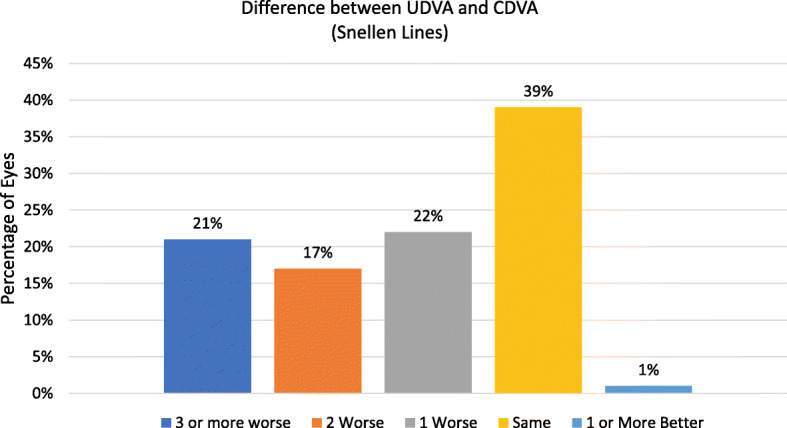
Histogram of lines of difference between postoperative UDVA and CDVA

The mean follow up time after bilensectomy was 14.5 ± 5.6 months. The mean age at bilensectomy was 44.4 ± 7.3 years. The most frequent cause for bilensectomy was cataract development (84 eyes), followed by corneal edema (1 eye), IOL dislocation (1 eye), refractive surprise (1 eye). Anterior subcapsular opacification was the most common type of cataract (43%) followed by the posterior subcapsular opacification (Figs. 1 and 2). Of the three models of pIOLs explanted, 72 were ICL (models V3 and V4, and one V4c), 7 were IPCL and 8 were PRL. The mean time between the pIOL implantation and bilensectomy was 85.2 ± 61.59 months. Table 1 shows the mean time between the implantation of each type of PC pIOL model and their subsequent bilensectomy. The preoperative and postoperative outcomes are presented in Table 2. The mean preoperative and postoperative UDVA and CDVA of each pIOL model are presented in Table 3. Sixty-eight percent of the patients had an UCVA of 20/40 or better, and 86% had a CDVA of 20/40 or better (Fig. 3). Thirty nine percent of the patients had the same uncorrected and corrected visual acuities postoperatively (Fig. 4). None of the eyes had a postoperative CDVA worse than the preoperative.

**Table 1:** Time in months between phakic IOL implantation and bilensectomy

Phakic IOL model	Time Mean ± SD
ICL	99.3 ± 43.7
IPCL	5.5 ± 2.7
PRL	67.1 ± 52.1

*ICL=* Implantable Collamer Lens, *IPCL=* Implantable Phakic Contact Lens, *PRL=* Phakic Refractive Lens

**Table 2:** Preoperative and postoperative visual and refractive results

	Preoperative Mean ± SD	Postoperative Mean ± SD	*P* value
UCVA (logMAR)	0.88 ± 0.63	0.31 ± 0.28	0.00
Sphere (D)	−0.63 ± 2.6	0.66 ± 1.1	0.00
Cylinder (D)	− 0.92 ± 1.08	−0.94 ± 0.84	0.71
Spherical equivalent (D)	− 1.10 ± 2.5	0.20 ± 1.2	0.00
CDVA (logMAR)	0.43 ± 0.44	0.15 ± 0.19	0.00
Endothelial cell density (cells/mm^2^)	2212 ± 667	2168 ± 618	0.67

*SD=* standard deviation, *UCVA=* uncorrected distance visual acuity, *CDVA=* corrected distance visual acuity

**Table 3:** Mean preoperative and postoperative UCVA, CDVA of each pIOL model

pIOL model	UCVA preoperative	UCVA postoperative	*P* value	CDVA preoperative	CDVA postoperative	*P* value
ICL (n: 72)	20/175	20/40	0.00	20/50	20/25	0.00
IPCL (n: 7)	20/80	20/25	0.01	20/35	20/20	0.01
PRL (n: 8)	20/60	20/30	0.08	20/50	20/25	0.04

*pIOL=* phakic intraocular lens, *UCVA=* uncorrected distance visual acuity, *CDVA=* corrected distance visual acuity

**Fig. 5:**
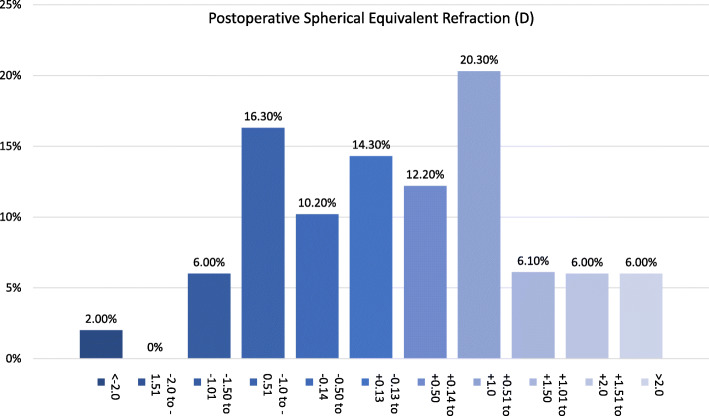
Postoperative spherical equivalent refraction

**Fig. 6:**
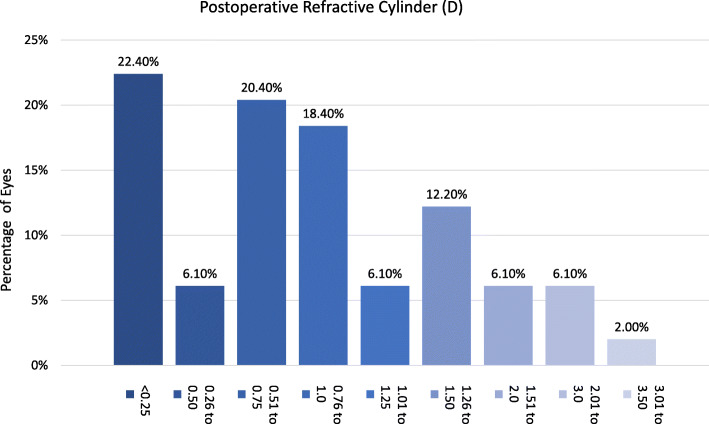
Postoperative refractive cylinder

The efficacy index was 0.8 (mean UDVA postop/ mean CDVA postop), 73% of the eyes were within ±1.00 D (spherical equivalent) of the attempted correction (Fig. 5). Regarding the refractive cylinder, 28.5% of the eyes had a postoperative value < 0.5 D (Fig. 6). Endothelial cell density did not significantly change after surgery (*p* = 0.67).

**Fig. 7:**
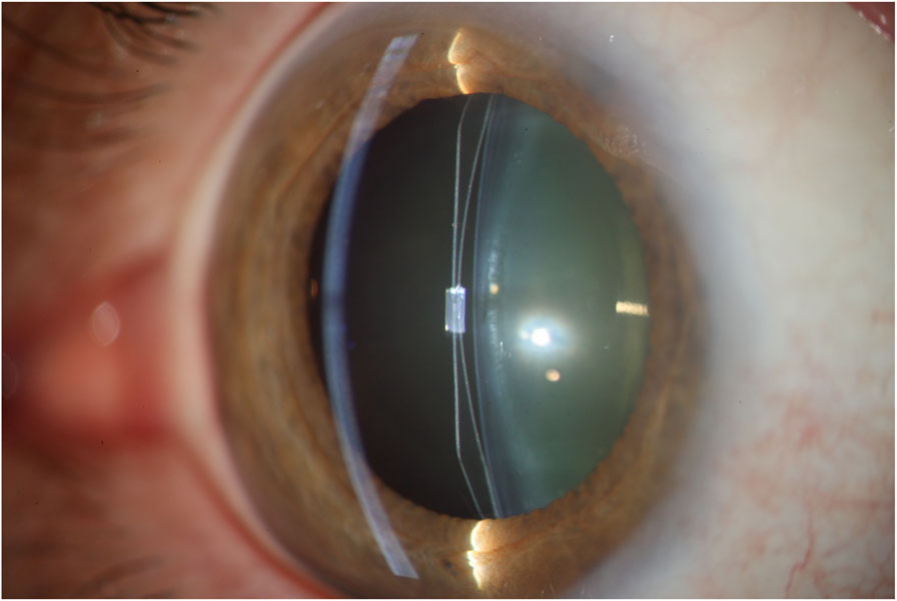
Posterior chamber phakic intraocular lens

Two eyes required a combined bilensectomy with trabeculectomy, postoperatively the IOP was controlled with a good final visual outcome (CDVA of 20/35). One eye had a combined bilensectomy with pupilloplasty with a final CDVA of 20/25, and one eye had a combined bilensectomy with Descemet’s stripping automated endothelial keratoplasty (DSAEK).

We had one case with pIOL (PRL) dislocation secondary to broken zonules, this patient did not present with any intra or postoperative complications. His final CDVA was 20/25.

Thirteen multifocal IOLs were implanted, all the rest were monofocal IOLs. The multifocal IOLs implanted were: Lentis Mplus LS 313 MF + 1.5D and + 3.0D (Oculentis GmbH, Berlin, Germany), AT Lisa tri 389 MP (Carl Zeiss Meditec, Henningsdorf, Germany), FineVision (PhysIOL SA, Liège, Belgium), ReSTOR AD1 (Alcon Lab, Fort Worth, Texas, USA), Versario Multifocal 3F IOL (Valeant Med Sp.zo.o., Warsaw, Poland) and AcriLISA 366D Carl Zeiss Meditec, Jena, Germany).

The visual outcome of these patients was good with a mean UDVA of 20/35, CDVA of 20/25 and an efficacy index of 0.8. Forty six percent of the patients implanted with a multifocal IOL were younger than 45 years, none of them had any postoperative optical complication.

Intraoperative complications were as follows: one eye had a posterior capsule rupture with the IOL implanted in the sulcus, 3 eyes required the use of pupil expanders for adequate pupil dilation.

Postoperative complications were as follows: as an early complication, one eye had a retinal detachment (RD) immediately after the surgery, eventually attaining a final CDVA of 20/130 after pars plana vitrectomy, this visual outcome was still better than the one he had before the bilensectomy (counting fingers).

As a late complication, four eyes presented with posterior capsule opacification that was successfully treated with neodymium-doped yttrium-aluminum-garnet (Nd:YAG) laser capsulotomy.

## Discussion

Refractive surgeons should consider the explantation of all phakic IOLs at some point [12]; either due to the incidence of cataract after the implantation of PC pIOLs [13, 14] or the development of an age-related cataract, a bilensectomy will be necessary in those patients, therefore it is important to report the long term outcomes of such procedures.

We found a significant improvement in both UDVA and CDVA after bilensectomy, with an acceptable efficacy index and good safety that correlates to the findings from other studies [9, 10]. Eighty six percent of our patients had a final CDVA of 20/40 or better. This apparently limited visual outcome is influenced by the concomitant retinal comorbidities of some of these high myopic patients such as previous RD (one eye had a RD one year after the implantation of the pIOL), foveoschisis, and myopic chorioretinal atrophy - all complications connected to high myopia [15].

Intra and postoperative complications were few, although one of them was sight-threatening (RD) and directly connected to the high myopia suffered by these patients.

Refractive outcomes were good, with a significant improvement in the sphere and spherical equivalent. The SRK-T formula with the Wang/Koch adjustment had good results, although nowadays the Barret Universal II formula is more accurate for the IOL calculation in cases with extreme myopia (AL > 28 mm) [16]. The different methods that can be used for IOL calculation in the presence of a pIOL are: standard ultrasound biometry, intraoperative ultrasound biometry, intraoperative autorefraction and partial coherence interferometry [17]. We used the latter as it provides adequate measurements in eyes with pIOLs [18].

The cylinder did not change after the bilensectomy; our outcomes agree with those reported in a previous study [9].

In a previous study [19], as well as in this study, cataract was the main cause for phakic IOL explantation. Anterior subcapsular opacification is the most common type of cataract after the implantation of a pIOL and it is presumed to be caused by the contact between the crystalline lens and the pIOL, trauma during surgery, intermittent trauma from accommodation, subclinical inflammation, insufficient vaulting, lens malnutrition and crystalline lens trauma from preoperative Nd:YAG laser peripheral iridotomy [6, 20]. The development of cataract with the latest model of ICL (V4c) is less common than with older models [7] due to the central hole that reduces the risk of cataract formation. The ICL (Fig. 7) was the most explanted PC pIOL, which is explained by the fact that it is the most widely implanted PC pIOL [21].

The time between PC pIOL implantation and bilensectomy differ depending on the pIOL model: the mean time was 8.2 ± 3.3 years for the ICL, which corroborate with the results from Meier et al. [9], but not with the ones from Kamiya et al. [10] (3.6 years). We assume that the difference in time was due to the ICL model, as Kamiya et al. [10] explanted more V2 models, which had a higher rate of cataract formation [22]. The mean time between the IPCL implantation and bilensectomy was short (0.4 years) compared to the other two PC pIOL models. A study [23] reported cataract formation within 1 year of IPCL implantation in 2.9% of the eyes studied, although only one eye required bilensectomy, with good final visual outcome (CDVA 20/25).

The PRL has been phased out the market due to its associated complications [5, 24]. It has been reported that contact with the haptics of the PRL causes zonular weakening which results in the dislocation of the pIOL [25]. Furthermore, patients with high axial myopia have weaker zonules due to the excessive stretching of the zonular fibers [24], and both factors might have contributed to the dislocation of the pIOL in our patient.

We had one combined procedure of bilensectomy and DSAEK secondary to endothelial cell loss; a study [26] reported good visual outcomes and graft survival in eyes undergoing this combined procedure. Endothelial cell loss rate after PC pIOL implantation differs between clinical studies [27–29]. It has been reported that there is no chronic loss of endothelial cells after the implantation of a PC pIOL [30], because there is no direct contact between the pIOL and the corneal endothelium. On the other hand, an 8-year follow up study [31] reported a mean percentage of endothelial cell loss of 6.2% after the implantation of the ICL pIOL.

We did not observe a significant loss in ECC after bilensectomy, so the procedure does not seem to significantly damage the corneal endothelium.

High myopic eyes and young patients (< 50 years) have a higher risk of RD [31–33] after cataract surgery. There was one case of RD in our study. This finding does not agree with previous studies [9–11] where no cases of RD were reported after PC pIOL bilensectomy. This might be secondary to our higher number of patients and the fact that our patients were younger (mean age of 44.4 years) than the patients in other studies [9, 10] (mean age of 47.2 and 50.39 years).

One eye required pupilloplasty due to a hyporeactive mydriatic pupil. Although pupillary defects are not common after the implantation of PC pIOLs, cases of fixed mydriatics pupils secondary to Urrets-Zavalia syndrome have been reported [34, 35]. Probably, pupil-related visual problems are an under reported feature in PC pIOL implantation.

Two eyes required a combined procedure with trabeculectomy: these eyes had pigment over the trabecular meshwork and high intraocular pressure. Sanchez-Galeana et al. [36] reported a case of intractable pigmentary glaucoma that required pIOL explantation and trabeculectomy in order to control the IOP. Pigment dispersion is related to chronic chafing by the pIOL.

One of the main limitations of this study is that we could not get the vault measurement of the PC pIOL before the bilensectomy surgery to ascertain its potential correlation with the cataract development.

To the best of our knowledge, this is a study with the largest number of eyes and the longest follow up time (mean 14.5 months) after PC pIOL bilensectomy, and the only one that reports the outcomes of three different types of PC pIOLs.

## Conclusions

In conclusion, the main cause for bilensectomy following PC-pIOL implantation was cataract development in our sample, and the visual and refractive outcomes were acceptable. It was a safe procedure in which we did not observe significant endothelial cell loss, and with few intra or postoperative complication rates. RD is a serious postoperative complication that should be monitored in young patients.

## Data Availability

The datasets used and/or analyzed during the current study are available from the corresponding author on reasonable request.

## Footnotes

1 IBERIA Biobank is a Collection of Intraocular Lenses and Other Ocular Explanted Devices in Spain and Portugal.
